# Early postoperative imaging after non-bariatric gastric resection: a primer for radiologists

**DOI:** 10.1007/s13244-017-0559-0

**Published:** 2017-06-19

**Authors:** Massimo Tonolini, Elena Bracchi

**Affiliations:** Department of Radiology, “Luigi Sacco” University Hospital, Via G.B. Grassi 74, 20157 Milan, Italy

**Keywords:** Gastrectomy, Complications, Anastomotic leakage, Contrast fluoroscopy, Computed tomography (CT)

## Abstract

**Abstract:**

Surgical resection represents the mainstay treatment and only potentially curative option for gastric carcinoma, and is increasingly performed laparoscopically. Furthermore, other tumours and selected cases of non-malignant disorders of the stomach may require partial or total gastrectomy. Often performed in elderly patients, gastric resection remains a challenging procedure, with significant morbidity (14–43% complication rate) and non-negligible postoperative mortality (approximately 3%). This paper provides an overview of contemporary surgical techniques for non-bariatric gastric resection, reviews and illustrates the expected postoperative imaging appearances, common and unusual complications after partial and total gastrectomy. Albeit cumbersome or unfeasible in severely ill or uncooperative patients, contrast fluoroscopy remains useful to rapidly check for anastomotic patency and integrity. Currently, emphasis is placed on multidetector CT, which comprehensively visualizes the surgically altered anatomy and consistently detects complications such as anastomotic leaks and fistulas, duodenal stump leakage, afferent loop syndrome, haemorrhages, pancreatic fistulas and porto-mesenteric venous thrombosis. Our aim is to help radiologists become familiar with early postoperative imaging, in order to understand the surgically altered anatomy and to differentiate between expected imaging appearances and abnormal changes heralding iatrogenic complications, thus providing a consistent basis for correct choice between conservative, interventional or surgical treatment.

***Teaching points*:**

• *Radical gastrectomy is associated with frequent postoperative morbidity and non-negligible mortality.*

• *In cooperative patients fluoroscopy allows checking for anastomotic patency and leaks.*

• *Multidetector CT with / without oral contrast comprehensively visualizes the operated abdomen.*

• *Awareness of surgically altered anatomy and expected postoperative appearances is warranted.*

• *Main complications include anastomotic and duodenal leaks, haemorrhages and pancreatic fistulas.*

## Introduction

Worldwide, gastric carcinoma (GC) represents one of the leading cancers and accounts for over 900,000 new cases and 723,000 deaths yearly [1]. GC shows dramatic geographic variation, with the highest incidence in China and Japan, parts of South America, Eastern Europe and Russia. Conversely, in North America and Europe this lethal disease has significantly declined because of improved nutrition, food refrigeration, eradication of *Helicobacter pylori* infection, decreasing tobacco use and alcohol intake. Whereas in the far East early GC detection through screening programs generally allows curative treatment, in the rest of the world most GCs are diagnosed at an advanced stage, resulting in dismal prognosis (27% 5-year survival rate) [1].

Resection with negative surgical margins represents the mainstay treatment and only potentially curative option for localized GCs, combined with adjuvant chemotherapy or chemo-radiotherapy for disease stages IB and higher. Radical gastrectomy remains a challenging surgical procedure with significant postoperative morbidity (14–43% complication rate) and mortality (0.8–12%, mean 3%) [2–6].

Traditionally, early post- gastrectomy imaging was limited to contrast fluoroscopy (CF) to assess anastomotic patency and integrity. More recently, multidetector CT is increasingly adopted to investigate most postoperative abdominal conditions, to comprehensively visualize the surgically altered anatomy and detect iatrogenic complications. However, compared to the literature about bariatric surgery imaging [7–9], few reports describe CT techniques and expected and abnormal appearances shortly after radical gastrectomy [10–12].

This paper provides an overview of contemporary surgical techniques, reviews and illustrates the expected postoperative imaging findings and complications after partial and total gastrectomy, aiming to provide radiologists with an increased familiarity in the interpretation of early post-gastrectomy CT studies and, ultimately, to limit iatrogenic morbidity.

## Overview of gastrectomy techniques

Surgical exploration and aggressive resection of GC with curative intent is undertaken unless medical contraindications exist or preoperative imaging shows metastatic spread or major vascular invasion. Surgical approaches depend on tumour site and extension: tumours of the cardia, proximal and mid-body stomach require total gastrectomy with removal of the entire stomach along with terminal oesophagus and proximal duodenum. Conversely, GCs of the distal body and antrum may be treated by partial gastrectomy, with stapling and division of the stomach at least 6 cm from any macroscopic tumour [2–5, 13].

Radical gastrectomy is completed with splenectomy and partial pancreatectomy in 17.5% and 7.1% of patients, respectively. The extent of lymphadenectomy remains controversial: compared to standard nodal dissection of perigastric nodes along the lesser and greater curvature, D2 lymphadenectomy is extended along the celiac axis, left gastric, common hepatic and splenic arteries. Gastric resection is sometimes performed to treat uncommon stomach malignancies (gastrointestinal stromal tumours, lymphomas or sarcomas) and selected benign conditions such as refractory peptic ulcers. Very recently, oncologic gastric surgery has also been performed using laparoscopy, resulting in decreased intraoperative blood loss and shorter hospital stays [2–5, 14, 15].

**Fig. 1:**
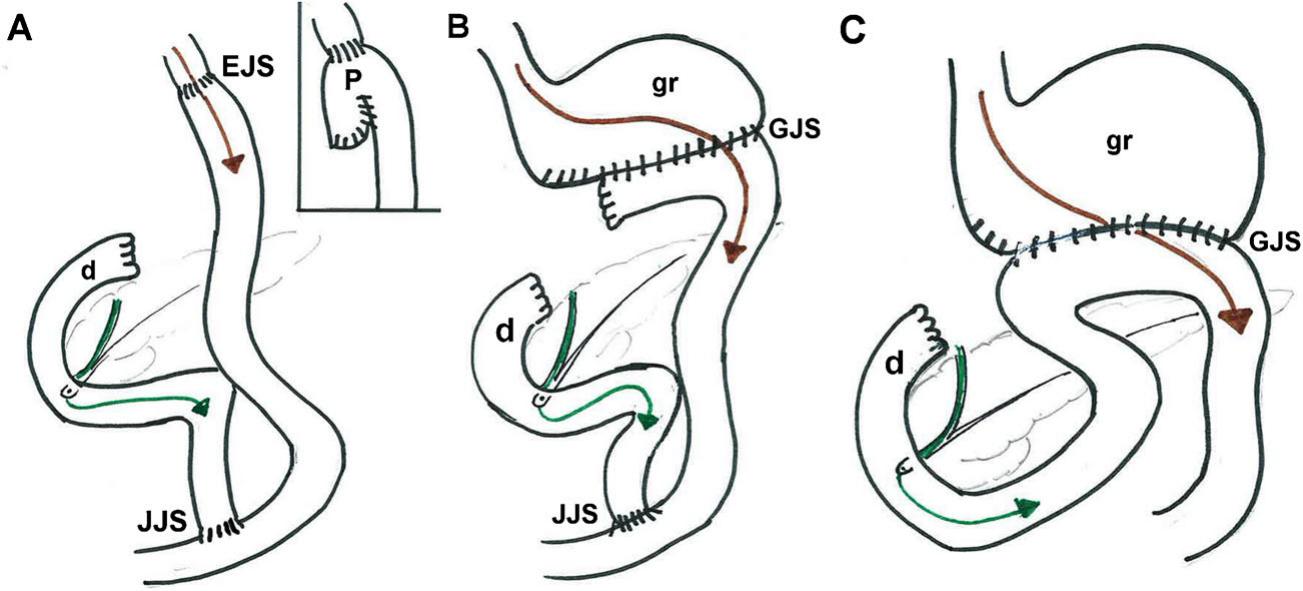
Schematic representations of post-surgical anatomy: A) after total gastrectomy with Roux-en-Y esophago-jejunostomy (EJS), jejuno-jejunostomy (JJS) and blind-ending duodenal stump (d); alternative technique with pouch (P) creation. Alimentary flow indicated by brown arrow, bile flow in dark green. B) after subtotal gastrectomy with small-sized gastric remnant (gr) connected to Roux-en-Y gastrojejunostomy (GJS). C) after partial gastrectomy reconstructed with a Billroth II GJS

Following gastrectomy, the choice of digestive tract anatomic reconstruction depends on the extent of resection. Most total gastrectomies are reconstructed with Roux-en-Y esophagojejunostomy (EJS), in which a jejunal limb is brought up and re-anastomosed to the distal oesophagus and to the closed duodenal stump (DS) (Fig. 1a). Whereas in “high” subtotal gastrectomy the small-sized gastric remnant is connected to a Roux-en-Y gastrojejunostomy (GJS) (Fig. 1b), “low” partial gastrectomy with preservation of the gastric fundus may be reconstructed using a Billroth II GJS (Fig. 1c) technique, with DS directly connected to the remnant stomach. According to the surgeon’s preference, anastomoses may be either hand-sewn or stapled [5, 13, 16].

## What to expect after radical gastrectomy

The likelihood of postoperative morbidity is strongly influenced by extended operation time, which acts as a surrogate of challenging surgical manoeuvres. No significant differences in complication rates exist with regard to resection extension between total, proximal or distal gastrectomy. Compared to open surgery, laparoscopic and laparoscopic-assisted techniques are not associated with significantly different morbidity, particularly concerning pulmonary complications, anastomotic leakage and intra-abdominal abscesses. Conversely, splenectomy and pancreatectomy represent risk factors for additional morbidity [2–5, 14, 15]. The effect of extended lymphadenectomy remains controversial: several studies showed that D2 nodal dissection is associated with higher postoperative morbidity, reoperation rates and mortality, specifically with increased likelihood of pulmonary complications, wound infection, failed anastomosis and pancreatic disorders [17, 18].

Similarly to other major abdominal surgeries, respiratory problems including pleural effusion, atelectasis and pneumonia commonly occur after oncologic gastrectomy, particularly in elderly men with chronic obstructive lung disease [4, 19].

The most important specific post-gastrectomy complications include anastomotic and DS leakage, pancreatic fistula, acute pancreatitis and intra-abdominal haemorrhage, and ultimately require reintervention in 25% of cases, particularly because of leakage, abscesses and bleeding. Anastomotic complications are most feared and account for most of septic morbidity and mortality [2–6, 20].

The commonest manifestations of intra-abdominal complications include physical and laboratory (leukocytosis, increasing C-reactive protein levels) signs of sepsis within the first 7 to 10 postoperative days (PODs). Bleeding is generally heralded by hypotension, dropping haematocrit, blood from nasogastric or drainage tubes. Most surgeons increasingly feel that abdominal pain and physical findings are relatively insensitive and increasingly rely on early postoperative imaging. When discussing indication and timing of post-surgical studies, asking the surgeon to draw the reconstructed anatomy generally proves helpful to interpret fluoroscopic and CT appearances [4, 21].

## Early post-gastrectomy imaging: techniques and normal appearances

### Contrast fluoroscopy

Fluoroscopic examination after oral administration of water-soluble CM has been largely adopted to check for possible anastomotic leakage. Albeit several institutions routinely perform CF within five PODs, testing of anastomosis should currently be obviated in asymptomatic patients [22, 23]. According to radiologist’s preference, either low-osmolar iodinated CM or diatrizoate meglumine may be used, but the latter is hyperosmolar and may induce pulmonary oedema if aspirated [24, 25].

**Fig. 2:**
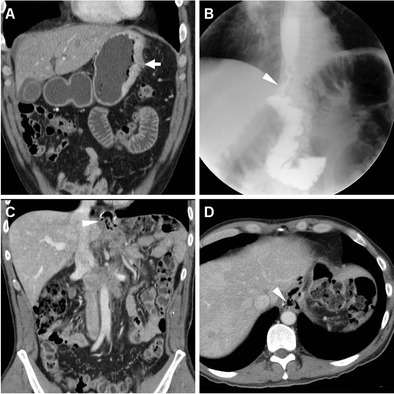
In a 39-year-old male with endoscopic and bioptic diagnosis of stomach adenocarcinoma, preoperative staging CT with oral water distension (A) showed marked solid, enhancing mural thickening from the gastric fundus along the greater curvature (thick arrow). On the fifth postoperative day (POD) after uncomplicated total gastrectomy with lymphadenectomy and splenectomy, contrast fluoroscopy (CF, B) showed normally patent, thin EJS (arrowhead) with opacification of jejunal limb and no anastomotic contrast medium leakage. Corresponding expected postsurgical contrast-enhanced CT (C, D) appearances at hospital discharge included stapled EJS (arrowheads) without peri-anastomotic air or fluid, absent spleen without collections in the surgical site

**Fig. 3:**
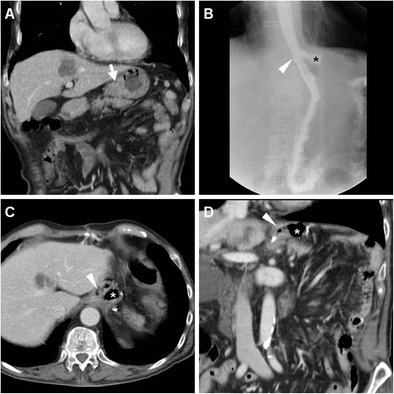
In a 74-year-old male, preoperative CT (A) depicted a stricturing carcinoma of the gastric body (thick arrow). Six days after uncomplicated total gastrectomy with lymphadenectomy and splenectomy, CF (B) visualized normally patent EJS (arrowhead) with a seemingly extraluminal contrast medium accumulation (*) which corresponded to anastomotic recess of the Roux-en-Y EJS at surgical correlation, a finding which potentially mimics leakage if technical details are not known. Corresponding expected postsurgical CT changes including air-filled anastomotic recess (*), non-stapled EJS (arrowheads) with unremarkable perianastomotic fat planes, no abnormal collections in the splenectomy site

**Fig. 4:**
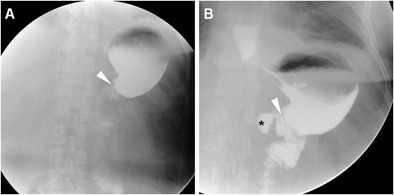
In a 62-year-old male, CF performed five days after uncomplicated partial gastrectomy showed opacified, moderately dilated remnant stomach, slow transit of iodinated contrast through the GJS (arrowheads) and opacified Roux-en-Y anastomotic recess (*)

**Fig. 5:**
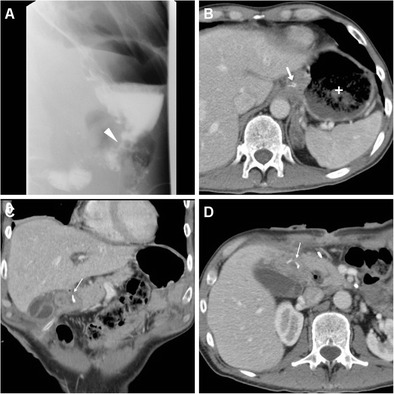
A 63-year-old male underwent partial gastrectomy with Billroth II reconstruction to relieve a large, non-malignant pyloric ulcer refractory to medical therapy. Early postsurgical CF (A) showed mildly delayed transit of iodinated contrast through the GJS (arrowhead), with fluid level in the gastric remnant. Corresponding CT (B-D) appearances before discharge included dilated remnant stomach (+) with ingested material, surgical staples at the gastric resection site (arrow in B), expected imaging appearance of the duodenal stump (DS, thin arrows in C, D) indicated by a single metallic staple, without surrounding fluid or collections

**Fig. 6:**
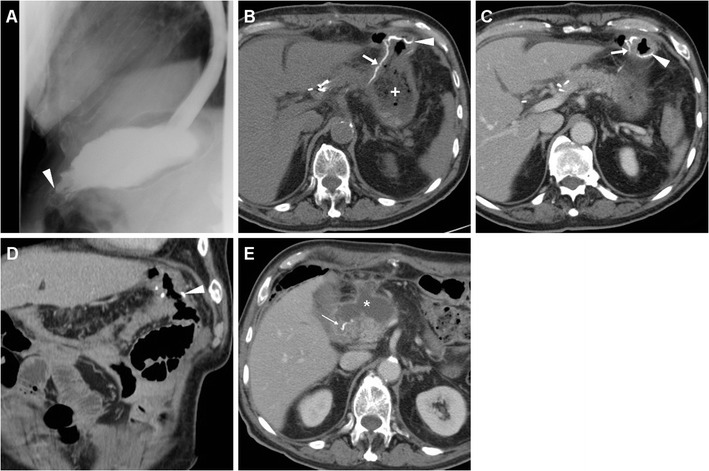
A 78-year-old male with antral gastric cancer underwent subtotal gastrectomy with D2 lymphadenectomy and Billroth II reconstruction and suffered from postoperative vomiting and fever. On 5th POD CF (A) showed limited, slow transit through the GJS (arrowhead). On 9th POD, unenhanced (B) and post-contrast (C-E) CT showed stapled gastric resection site (arrows) and GJS (arrowheads), minimally dilated remnant stomach (+), and a fluid collection (* in E) abutting the DS (thin arrow) consistent with DS leakage, which was successfully managed conservatively but required prolonged hospitalization

Rapid sequences in frontal and oblique views are acquired during ingestion or sipping of CM via a straw, with the patient standing or sitting on the fluoroscopy table: unfortunately, CF is very cumbersome when the recently operated patient is unable to cooperate or cannot swallow [24]. Compared to the EJS, which is generally recognized at the level or immediately below the diaphragmatic hiatus (Figs. 2 and 3), the position of a GJS is more variable and requires higher degrees of left- or right-sided patient rotation (Figs. 4, 5 and 6).

**Fig. 7:**
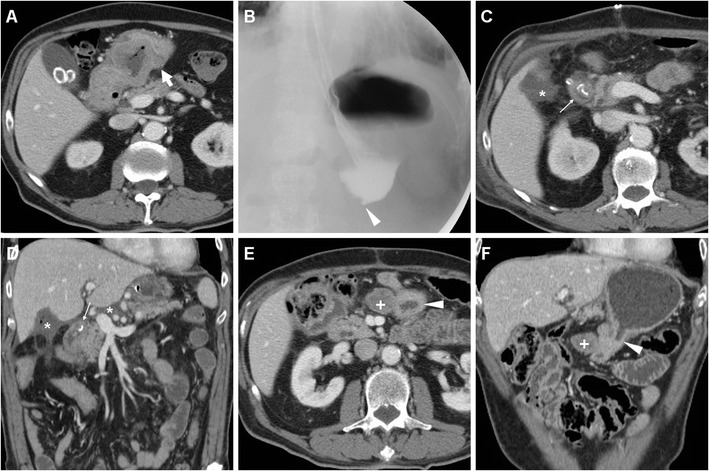
A 76-year-old male with stricturing antro-pyloric carcinoma (thick arrow) as preoperatively depicted by CT (A) underwent subtotal gastrectomy with Roux-en-Y reconstruction. Early postoperative CF (B) showed little or no transit through the GJS (arrowhead). On 8th POD during postoperative sepsis, contrast-enhanced CT (C, D) showed normal stapled DS (thin arrows), minimal fluid (*) in the cholecystectomy site. The patient then improved on conservative therapy including gastric intubation. Subsequent endoscopy showed ischaemic ulceration of the GJS. Repeated CT (E, F) three months later showed circumferential mural thickening of the GJS (arrowheads) and perianastomotic nodule (+) consistent with tumour recurrence

After uncomplicated partial gastrectomy, delayed CM passage through the GJS, incomplete emptying and fluid-fluid level in the gastric remnant are fairly common, most usually self-limiting and secondary to ileus or anastomotic oedema, and generally do not represent a complication (Fig. 4); however, in our experience anastomotic ulceration may have a similar appearance and may ultimately result in stricture (Fig. 7) [5].

**Fig. 8:**
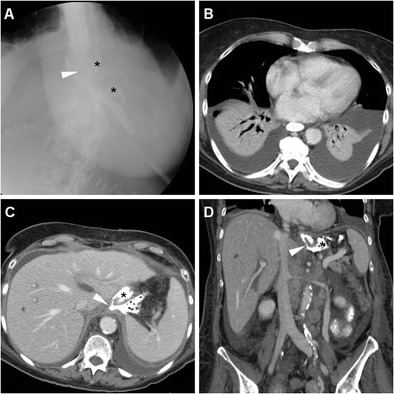
In a 69-year-old woman with advanced carcinoma of the gastric body, palliative total gastrectomy was performed. Five days after surgery, CF (A) showed patent EJS with opacification of extensive anastomotic leakage (*). A few days later, on conservative management, contrast-enhanced CT (B-D) showed bilateral pleural effusions and atelectasis (B), patent EJS (arrowheads) and persistent leak (*)

Albeit perianastomotic extravasation (Fig. 8) is nearly 100% specific for leakage, a pitfall of CF is represented by misinterpretation of the anastomotic recess of an end-to-side reconstruction as extraluminal CM (Figs. 3 and 4) [12].

### Early post-gastrectomy CT

Nowadays, in most post-surgical situations, multidetector CT rapidly and consistently provides panoramic visualization of postoperative abdominal changes, and generally adds crucial information for diagnosis of iatrogenic complications [10, 11, 26].

Due to high prevalence of pleuropulmonary changes (Fig. 8), the CT acquisition should encompass the entire abdomen and lung bases. Some authors reported that early post-gastrectomy CT without oral CM is easier, faster and safer to perform shortly, particularly in bed-ridden uncooperative patients. Precontrast scans and lung or viewing with bone window settings easily allow identifying metallic surgical staples and intraperitoneal air, and differentiating between fluid and haemorrhagic collections. Unless contraindicated by allergy or renal failure, CM-enhanced acquisition, at least in the portal-venous phase, is warranted. When clinical or laboratory findings suggest possible postoperative bleeding, an additional arterial-phase scanning may allow detection of active haemorrhage. In our experience, CT review along axial, coronal and sagittal planes may be beneficial.

A useful checklist for CT interpretation is provided in Table 1. Postoperative pneumoperitoneum is a common, expected finding which typically distributes freely and resolves within a few days. On plain radiographs or CT, intraperitoneal air may last up to 9–10 PODs, but decreases on serial imaging. Conversely, persisting or increasing gas raises concern for anastomotic dehiscence or visceral perforation. Following laparoscopy, the amount of expected intraperitoneal air is less compared to open surgery, because insufflated CO2 is rapidly absorbed; conversely, subcutaneous emphysema may result from insufflation in the abdominal wall [26, 27].

**Table 1:** Checklist for interpretation of early post-gastrectomy CT

**Feature**	**Comments**
Report pleuropulmonary changes at lung bases	Atelectasis, pneumonia, pleural effusion (see Fig. 8) or empyema (Fig. 14)
Quantify free intraperitoneal air and/or fluid	Discussion in text; see Fig. 10
Identify stapled or hand-sewn - esophagojejunostomy (EJS) after total gastrectomy (Figs. 2 and 3) - gastrojejunostomy (GJS) after partial gastrectomy (Figs. 5 and 6) - look for localized air, fluid or haemorrhagic collections adjacent to either EJS or GJS (Figs.11 and 12)	Gastric remnant generally indicated by staple line Efferent jejunal limb from EJS or GJS characterized by valvulae conniventes and tubular configuration on coronal viewing
Identify the closed duodenal stump (DS) - assess dilatation (Fig. 15) - look for adjacent collections (Figs. 6 and 9)	DS generally recognized by a metallic staple at its blind end (Figs. 5, 6, 7 and 9)
Report presence and site of drainage tubes	
Assess post-splenectomy or post-pancreatectomy status	If performed (see Figs. 2, 3 and 11)
Assess patency of splenic, portal and mesenteric veins	For postoperative thrombosis, favoured by intra-abdominal sepsis
Exclude retained foreign bodies	E.g. surgical sponges (indicated by thin hyperattenuating structures), bioabsorbable haemostatic materials agents such as Gelfoam or Surgicel (which appear as walled heterogeneous masses with internal “mottled” gas bubbles)
Scrutinize laparotomy site	For abscess collections suggesting wound infection

**Fig. 9:**
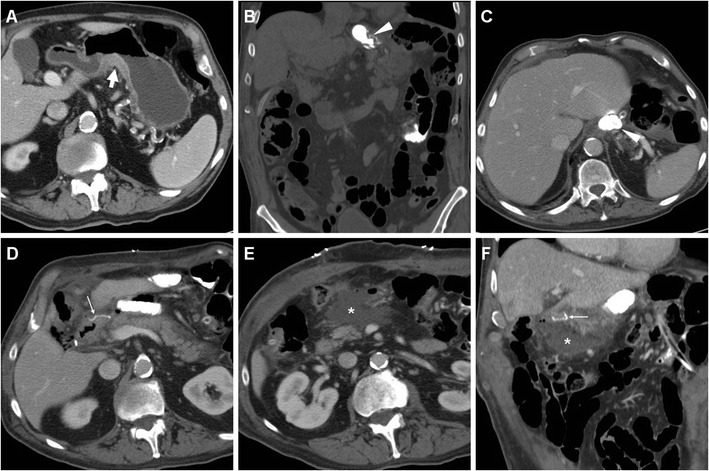
An 81-year-old male with T3 N2 cancer of the lesser gastric curvature (thick arrow) as preoperatively depicted by CT with oral water distension (A) underwent total gastrectomy with Roux-en-Y reconstruction. On 9th POD CT was performed because of bile from drainage: unenhanced (B) and post contrast (C-F) scans with oral contrast showed normally opacified and patent EJS (arrowheads) without evidence of anastomotic leak (note tip of surgical drainage abutting the EJS in C). Abutting the stapled DS (thin arrows) a non-capsulated fluid collection (*) corresponded to DS leakage which was conservatively managed

Borrowing from experience in bariatric surgery, oral administration of positive water-soluble CM may improve the CT performance, particularly regarding anastomotic leaks. If the patient can stand and swallow without risk of aspiration, we instruct him or her to drink low-osmolar CM such as 3% diluted iopamidol or iohexol 5–10 min prior to CT. Lack of extraluminal CM is strongly consistent with anastomosis integrity (Fig. 9). Finally, CT results strongly influence reoperation rate, mortality, duration of postoperative fasting and hospitalization [10–12].

## Anastomotic complications

### Anastomotic leaks

Leakage resulting from breakdown of a suture line secondary to either inadequate technique, excessive tension or ischaemia, occurs after 3–4% of gastrectomies with either hand-sewn and stapled anastomosis, generally as an early complication within the first PODs;. Incidence of leakage may reach 21% with routine use of CT. Albeit variably symptomatic, anastomotic dehiscence represents an independent predictor of survival with 10–20% associated mortality [2–4].

**Fig. 10:**
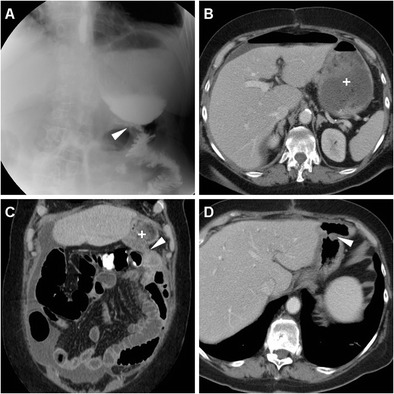
A 77-year-old female with distal gastric carcinoma underwent partial gastrectomy. Early postoperative CF (A) showed slow emptying of gastric remnant through the GJS (arrowhead), without appreciable extraluminal leaks. Six days later, the patient suffered from high fever: contrast-enhanced CT (B-C) showed dilated remnant stomach (+), absence of collections adjacent to the GJS (arrowheads in C and D), but persistent intraperitoneal air and fluid consistent with peritonitis, confirmed at surgical exploration: after redo anastomosis, the final status of GJS (arrowhead) is shown in D

The fluoroscopic hallmark of anastomotic leak is represented by CM extravasation adjacent to a GJS or EJS (Fig. 8), with either contained collection or free dispersal in the adjacent surgical cavity. However, since the reported sensitivity varies in the range 22% to 67%, negative CF findings do not rule out the possibility of leakage (Fig. 10) [22].

**Fig. 11:**
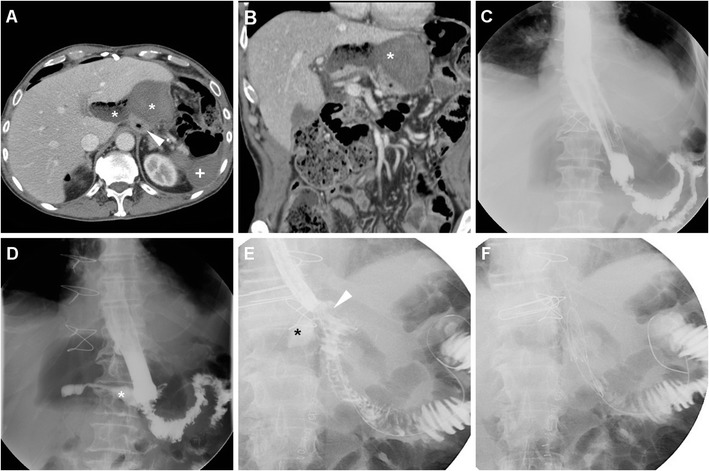
A 68-year-old male with recurrent gastric carcinoma on previous Billroth II gastrectomy for peptic ulcer was treated with total gastrectomy. Postoperative anemization required blood transfusions. On 12th POD, contrast-enhanced CT (A, B) showed persistent intraperitoneal air, a mixed attenuation collection (*) adjacent to the EJS (arrowheads), consistent with anastomotic dehiscence, a subacute blood collection (+) in the site of splenectomy. After percutaneous drainage, AL was treated with positioning of a metallic stent (C) through the EJS. Persistent leakage of oral iodinated contrast (*) at follow-up CF (D, E) led to removal of the stent and positioning of a self-expanding stent (F)

**Fig. 12:**
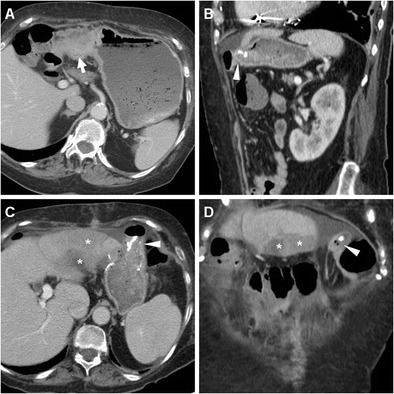
An 81-year-old male with advanced, obstructing antral cancer (thick arrow) as preoperatively depicted by CT (A) underwent palliative partial gastrectomy with Roux-en-Y reconstruction. During prolonged hospitalization the patient experience anemization and failure to thrive. Early CF (not shown) showed slow transit through EJS. On 11th POD, contrast-enhanced CT (B-D) showed some ingested materials in the remnant stomach (+), stapled EJS (arrowheads), some residual intraperitoneal air and fluid, inhomogeneous hypoattenuation of the 3rd liver segment consistent with parenchymal infection

As discussed above, the use of CT is favoured over CF in uncooperative patients. Even without oral CM, CT is felt to be superior to CF with 89.5% positive predictive value compared to 40%, mostly because CT shows both the anastomosis and the surrounding extraluminal compartment. On CT images the EJS or GJS should be carefully scrutinized for focal discontinuity and mural thickening. Since anastomotic dehiscence leads to the extravasation of enteral material in the surgical site, the most common appearance includes variably sized air-fluid collections, abscesses or inflammatory changes which abut the EJS or GJS (Figs. 11 and 12) and tend to extend into the lesser sac, left subphrenic, gastrohepatic and gastrosplenic spaces [10–12].

Alternatively, anastomotic leak may sometimes appear as generalized peritonitis with free fluid and intraperitoneal air (Fig. 10). Suspicious findings which support a diagnosis of visceral perforation over residual postoperative air in the peritoneal cavity include persistence or increase of pneumoperitoneum, particularly if abundant (over 20 cm^3^) and occupying a single compartment. It has been reported that persistent air after POD 5 associated with leukocytosis has 80% sensitivity for identifying patients requiring re-operation [26, 27].

**Fig. 13:**
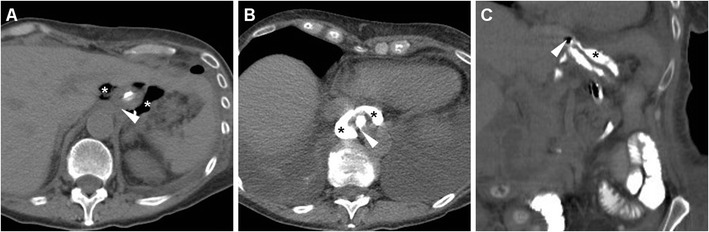
In a 76-year-old woman with T4 N2 gastric cancer, three days after total gastrectomy unenhanced CT (A) showed minimal residual pneumoperitoneum and extensive air (*) surrounding the EJS (arrowheads). Repeated unenhanced CT with oral contrast administration (B, C) showed patent EJS (arrowheads) and confirmed circumferential anastomotic leakage (*), which was initially managed conservatively but ultimately required reintervention with redo anastomosis six days later because of failed clinical improvement and progressive anemization

Albeit plain CT without enteral CM is rapidly performed even in critically ill patients, according to some reports positive oral CM improves the CT performance: leakage is heralded by extraluminal CM collecting nearby the EJS or GJS, or dissecting in the postoperative neocompartment (Figs. 8 and 13) [10–12, 22, 23].

**Fig. 14:**
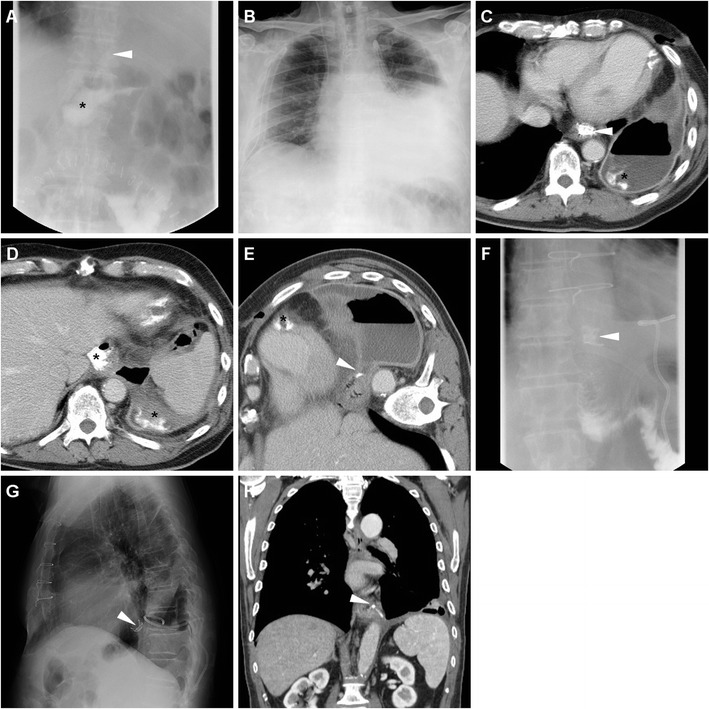
A 59-year-old male with cardial carcinoma underwent total gastrectomy. Early postoperative CF (A) showed patent EJS (arrowhead) with perianastomotic contrast leakage (*) directed medially. Bilateral pleural drainages were positioned to relieve minimal right pneumothorax and left hydropneumothorax (B). On 10th POD contrast-enhanced CT with oral contrast (C, D) confirmed ample left-sided hydropneumothorax with enhancing pleural surface. Extraluminal contrast leakage (*) was seen both medially to the EJS (arrowheads) and dependent in the left pleural empyema. Repeated CT obtained in the right lateral decubitus position (E) before CT-guided pleural drainage confirmed anastomotic fistula from the EJS (arrowhead) to the hydropneumothorax, which was treated by endoscopic clipping (arrowhead in F); note pleural pigtail drainage

In the past, diagnosis of anastomotic dehiscence warranted immediate surgical re-exploration with toilette, closure of leakage or redo anastomosis, placement of drainage tubes and enteral feeding. More recently, in stable patients with contained leaks and no peritonitis, non-operative management is increasingly considered: endoscopic stenting (Fig. 11) is becoming the preferred solution, and other options include endoscopic clipping (Fig. 14) and percutaneous drainage of fluid collections. Reoperation is currently reserved for wide dehiscence, peritonitis or failure of nonoperative management [4, 12, 23].

### Anastomotic fistulas

Well-known to radiologists, fistulas represent abnormal communications between the stomach and other structures such as the skin, bronchial tree or pleural cavity, which allow passage of enteral fluids. After gastrectomy, subphrenic infection secondary to anastomotic leak may occasionally be complicated by formation of gastro-cutaneous, gastro-bronchial or gastro-pleural (Fig. 14) fistulas, which represent rare occurrences described in sparse case reports. Albeit CF studies may show abnormal passage of CM through the fistula, CT more effectively depicts associated changes and consequences such as subphrenic abscess, pneumonia or empyema (Fig. 14) [10, 11].

### Anastomotic ulcers and strictures

Anastomotic ulcers may develop after gastrectomy, but are unreliably depicted by imaging compared to endoscopy. Ulcers plus ischemia and scar formation may ultimately lead to the formation of an anastomotic stricture at the GJS (Fig. 7). Anastomotic strictures develop in up to 3–4.4% of operated patients, generally present weeks or months after surgery and manifest with nausea, vomiting, dysphagia or post-prandial pain [3].

## Duodenal stump complications

### Afferent loop syndrome

**Fig. 15:**
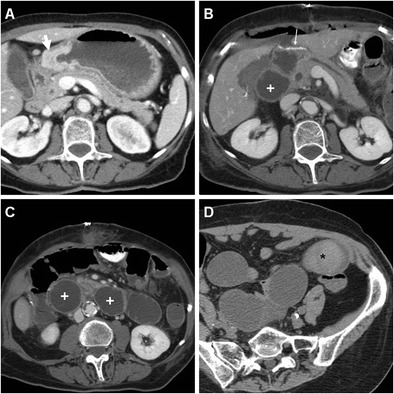
A 72-year-old female with distal gastric carcinoma (thick arrow) as preoperatively depicted by CT (A) underwent total gastrectomy. On 6th POD the patient suffered from persistent abdominal pain and anemization. Postoperative CT (B-D) showed dilated fluid-filled duodenum (+) up to stapled DS, consistent with afferent limb syndrome, secondary to a large blood clot in the jejunum which was best seen as a hyperattenuating intraluminal mass (*) on precontrast (D) scans. Surgical reintervention was required to relieve obstruction

The uncommon afferent loop syndrome generally occurs after Billroth II reconstruction from obstruction of the afferent blind DS by adhesions, internal hernia, intraluminal blood or bezoar, anastomotic kinking, ulcer or stricture, and is heralded by fluid-filled duodenal dilatation (Fig. 15) [10, 11].

### Duodenal stump leakage

A potentially worrisome (6–11.7% mortality) complication of Billroth II reconstruction, DS leakage occurs after 3% of partial gastrectomies and corresponds to the spread of bile from the blind-ending DS because of technical failure, ischemia or distal obstruction, causing local and ultimately peritoneal irritation [28]. Usual CT appearance includes a fluid collection abutting the stapled DS and extending to the right sub-hepatic or peripancreatic spaces (Figs. 6 and 9) [10, 11].

Currently, conservative treatment (parenteral nutrition, antibiotics, octreotide, suction drains and percutaneous drainage of abscesses) is successful in over 90% of cases; reoperation is reserved for septic patients or failure of conservative approach [4, 28].

## Vascular complications

### Haemorrhage

Blood loss after gastrectomy may represent a life-threatening complication and generally results from inadequate haemostasis, particularly ligation of small feeding vessels. As it occurs at a staple line or anastomosis, early postoperative haemorrhage is most usually intraluminal rather than in the surgical site or peritoneal cavity. Conversely, delayed bleeding is mostly due to marginal anastomotic ulcers. CT findings include intraluminal high-attenuation blood or clots in the bowel (Fig. 15), sometimes extravascular CM “blush” corresponding to active bleeding. The latter sign and endoscopically occult haemorrhage represent indications for transarterial embolisation, particularly in patients unfit for surgical reintervention [4, 29].

## Pancreatic fistula and pancreatitis

Occurring after 7.6% of all gastrectomies, postoperative pancreatic fistula corresponds to leaking pancreatic secretions and develops secondary to resection or injury to the pancreatic capsule. Fatty pancreas and pancreas divisum are the key risk factors. The clinical and laboratory diagnosis requires any measurable output from peripancreatic drainage on or after POD 3 with amylase content over three times the serum amylase; alternatively pancreatic fistula is diagnosed at percutaneous drainage or surgical reintervention [4, 30].

**Fig. 16:**
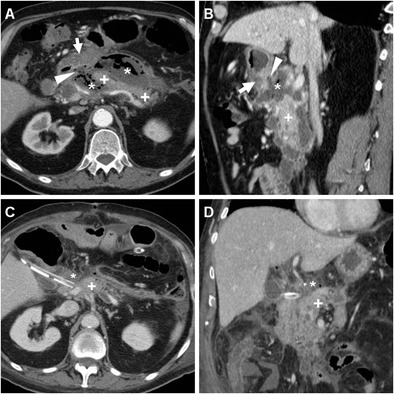
An 84-year-old male with lesser sac and pancreatic abscess (* in A, B) from retroperitoneal perforation of pyloric peptic ulcer (arrowheads) underwent emergency surgery, including subtotal gastrectomy with Billroth II reconstruction. Note pancreatic parenchyma (+), oedematous thickening of pylorus submucosa (thick arrows). On 7th POD surgical revision was required due to DS biliary leakage, with positioning of Kehr tube. Subsequently, on 20th POD follow-up CT (C, D) showed minimal fluid adjacent to the drainage tube, consistent with laboratory diagnosis of postoperative pancreatic fistula

At CT, the usual appearance is a focal fluid collection in the surgical bed, particularly in the lesser sac or adjacent to the pancreas (Fig. 16). Currently, postoperative pancreatic fistula is effectively managed nonsurgically until closure with parenteral nutrition and percutaneous drainage of dominant collections [31–33].

Alternatively, acute postoperative pancreatitis may develop in the gland remnant after partial pancreatectomy, and appears as segmental or diffuse enlargement with peripancreatic inflammatory changes and fluid collections extending to the retroperitoneal fasciae and the anterior pararenal space [4].

## Conclusion

Albeit CF may still be used as first-line imaging, multidetector CT after radical gastrectomy increasingly allows comprehensive visualization of the operated abdominal compartment and provides a consistent basis for correct choice between conservative, interventional or surgical treatment. In our experience, early post-gastrectomy CT benefits from oral CM administration if permitted by the patient’s conditions and cooperation. Understanding the surgically altered anatomy and knowledge of expected postoperative appearances is crucial to correctly recognizing complications.
